# Supplementary material for: Screening identifies small molecules that enhance the maturation of human pluripotent stem cell-derived myotubes
Source: eLife. 2019 Nov 11;8:e47970. doi: 10.7554/eLife.47970 (PMC6845233; doi:10.7554/eLife.47970)
Supplement: Figure 6—source data 1. [file elife-47970-fig6-data1.docx]

**Figure 6-source data 1**

**IPA of upstream regulators of the differentially expressed genes upon combinatorial treatment confirm the pathways targeted by the small molecules. Activation Z-scores <-2 and >2 are considered significantly inhibited and activated, respectively.**

| **Upstream regulators** | **Activation z-score** | **p-value of overlap** |
| --- | --- | --- |
| SB-431542 | 2.174 | 1.83E-14 |
| TGFB1 | -6.125 | 1.83E-40 |
| TGFB3 | -4.009 | 2.21E-21 |
| NOTCH1 | -2.646 | 1.39E-11 |
| NOTCH | -2.377 | 3.54E-07 |
| NOTCH3 | -1.558 | 9.41E-08 |
| Dexamethasone | 2.045 | 1.43E-27 |
| Forskolin | 2.819 | 4.82E-16 |
