# Supplementary material for: Screening identifies small molecules that enhance the maturation of human pluripotent stem cell-derived myotubes
Source: eLife. 2019 Nov 11;8:e47970. doi: 10.7554/eLife.47970 (PMC6845233; doi:10.7554/eLife.47970)
Supplement: Figure 6—source data 2. [file elife-47970-fig6-data2.docx]

**Figure 6-source data 2**

**List shows the targets of transcription factors and miRNA that were found differentially expressed upon combinatorial treatment.**

| **TEAD4** | **MYF6** | **KLF4** | **MEF2A** | **MEF2C** | **MIR-133** |
| --- | --- | --- | --- | --- | --- |
| *CKM*  *MYH1*  *TNNT2*  *SLC2A4*  *ACTA1*  *DES*  *TNNC1*  *SLC2A3*  *NOTCH2*  *PLK2*  *FLT1*  *CYR61*  *CTGF*  *BMP4* | *CKM*  *FGF6*  *CCND3* | *KDR*  *IRX3*  *FGF5*  *PRKG1*  *APCDD1*  *ACTC1*  *KIF16B*  *MYH6*  *ATP2A2*  *PPARA*  *SOD2*  *WLS*  *FARP1*  *CENPE*  *PCDH18*  *LAMA1*  *NRP1*  *EPHA2*  *TGFB2*  *ALCAM*  *TGFB1*  *PAX3*  *CD44*  *CCND2*  *FN1*  *ANGPT1*  *ID3*  *HES1*  *FLT1*  *BCL11B*  *PLAT*  *CITED1*  *TEK*  *SEMA3F*  *TAGLN*  *FRZB*  *ACTA2*  *COL8A1*  *MYOCD*  *SERPINE1*  *COL1A1*  *CYP26A1*  *ALDH1A1*  *CDKN2B*  *LGR5*  *TNC*  *KRT17*  *VCAM1*  *SERPINB2* | *CKM*  *PPARGC1A*  *NR4A1*  *SLC2A4*  *ENO3*  *ACTA1*  *XIRP2*  *ACTC1*  *TNNC1*  *MYH6*  *ATP2A2*  *TGFB2*  *TGFB1*  *BDNF*  *MYOCD*  *MMP10* | *BLK*  *CKM*  *PPARGC1A*  *TNNI2*  *ABRA*  *MYOZ1*  *LMOD2*  *MYH1*  *TNNT2*  *NR4A1*  *HSPB7*  *SLC2A4*  *mir-1*  *ATP2A1*  *SMYD1*  *MYOZ2*  *CASQ2*  *ITGB1BP2*  *mir-133*  *TTN*  *MYLPF*  *ACTA1*  *FOSB*  *DES*  *MYL4*  *KCNJ2*  *MYOM2*  *ACTN2*  *RYR2*  *ACTC1*  *MYOT*  *TNNI1*  *MEF2A*  *TNNC1*  *PHKA1*  *MYOM1*  *MYH6*  *ATP2A2*  *PPARA* | *CKM*  *PPARGC1A*  *NR4A1*  *SLC2A4*  *ENO3*  *ACTA1*  *XIRP2*  *ACTC1*  *MEF2A*  *TNNC1*  *MYH6*  *ATP2A2*  *TGFB2*  *TGFB1*  *BDNF*  *MYOCD*  *MMP10* |
